# Ocean Bottom Seismometer: Design and Test of a Measurement System for Marine Seismology

**DOI:** 10.3390/s120303693

**Published:** 2012-03-19

**Authors:** Antoni Mànuel, Xavier Roset, Joaquin Del Rio, Daniel Mihai Toma, Normandino Carreras, Shahram Shariat Panahi, A. Garcia-Benadí, Tim Owen, Javier Cadena

**Affiliations:** 1 SARTI Group, Electronics Department, Universitat Politècnica de Catalunya, UPC.Vilanova i la Geltrú 08800, Spain; E-Mails: antoni.manuel@upc.edu (A.M.); xavier.roset@upc.edu (X.R.); daniel.mihai.toma@upc.edu (D.M.T.); normandino.carreras@upc.edu (N.C.); shahram137@hotmail.com (S.S.P.); javier.cadena@upc.edu (J.C.); 2 Carrack Measurement Technology, Dullingham, Newmarket, Suffolk CB8 9UP, UK; E-Mail: timowen@carrack.co.uk

**Keywords:** ocean bottom seismometer, geophone, sensor modeling, refraction seismicity, clock synchronization, precision time protocol

## Abstract

The Ocean Bottom Seismometer (OBS) is a key instrument for the geophysical study of sea sub-bottom layers. At present, more reliable autonomous instruments capable of recording underwater for long periods of time and therefore handling large data storage are needed. This paper presents a new Ocean Bottom Seismometer designed to be used in long duration seismic surveys. Power consumption and noise level of the acquisition system are the key points to optimize the autonomy and the data quality. To achieve our goals, a new low power data logger with high resolution and Signal–to-Noise Ratio (SNR) based on Compact Flash memory card is designed to enable continuous data acquisition. The equipment represents the achievement of joint work from different scientific and technological disciplines as electronics, mechanics, acoustics, communications, information technology, marine geophysics, *etc.* This easy to handle and sophisticated equipment allows the recording of useful controlled source and passive seismic data, as well as other time varying data, with multiple applications in marine environment research. We have been working on a series of prototypes for ten years to improve many of the aspects that make the equipment easy to handle and useful to work in deep-water areas. Ocean Bottom Seismometers (OBS) have received growing attention from the geoscience community during the last forty years. OBS sensors recording motion of the ocean floor hold key information in order to study offshore seismicity and to explore the Earth’s crust. In a seismic survey, a series of OBSs are placed on the seabed of the area under study, where they record either natural seismic activity or acoustic signals generated by compressed air-guns on the ocean surface. The resulting data sets are subsequently used to model both the earthquake locations and the crustal structure.

## 1. Earthquake and Controlled Seismic Source—Comparison of Ocean Bottom Seismometers

Ocean Bottom Seismometers are used in earthquake seismology, where seismic energy generated by earthquakes and minor earth tremors is used as the source, and, more extensively, in active or controlled seismic source where an artificially generated sound source is used to provide the seismic energy. These two applications of OBSs have different instrument requirements because of the nature of the sources and differences in the nature of the information that can be obtained using the two approaches.

Earthquake seismology uses seismometers to record distant arrivals from a few large earthquakes or from more local swarms of smaller tremors—these arrivals can be used to give information relating to the source mechanisms and source area if enough stations record them, or data relating to the receiver locality, but only very diffused data relating to the intervening earth’s crust [1,2]. The signals generated by large earthquakes are of much lower frequency that those generated by the controlled sources, so that special sensors are required. Because of the technical requirements of a frequency response, sensitivity and signal to noise ratio, sensors suitable for earthquake seismology are usually much larger and heavier than those necessary for controlled seismic source, and therefore the constraints imposed on the OBS system are different. An earthquake OBS therefore needs to be designed to carry and deploy a heavier and larger sensor package suitable for recording from 0.01 Hz to about 30 Hz, and to operate autonomously for up to six months or one year duration. Because of the longer deployment durations, the timing requirements may necessitate a more accurate internal clock.

Controlled seismic source is aimed at obtaining higher resolution images of the upper layers of the earth’s crust than can be obtained by earthquake seismology. It typically uses many recording stations, often as close as 100 m, and many shots of similar or closer spacing using the same air gun arrays as are normally used in seismic reflection surveys. By achieving a uniform coverage of the target region it is possible to delineate structures and detect faults and track geological layers from one area to another. The frequency range of typical sources is in the region of 5 to 150 Hz, and controlled seismic source OBS recording systems cannot normally record below a few Hz. Within the field of controlled source there are widely different applications. In general, academic controlled source surveys tend to be large scale and concerned with broad structures and use relatively sparse receiving arrays of OBSs—typically a few tens of instruments. Commercial controlled source refraction surveys tend to be concerned with delineating smaller scale structures related to mineral resources, and use many hundreds of OBSs. The required number of instruments to be deployed avoids the use of free fall and acoustic released OBSs for large scale commercial surveys, thus, Remotely Operated Vehicles (ROVs) are used to deploy and recover instruments. The Ocean Bottom Seismometers described here were initially focused on small scale controlled seismic source or on earthquake seismology, but can have wider applications as a platform for seabed data collection.

**Figure 1.**
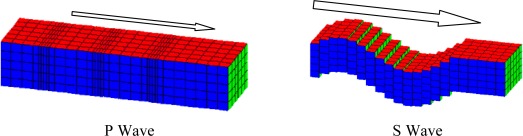
Primary and secondary waves.

Both earthquake and controlled seismic source use the propagation of both P (primary) and S (secondary) waves through the earth’s crust to obtain information relating to the propagation path. The S-wave moves as a shear or transverse wave, so motion is perpendicular to the direction of wave propagation, and P-wave has the highest velocity and is therefore the first to be recorded, and is formed from alternating compressions and rarefactions [3,4]. Figure 1 depicts how these waves propagate. P waves travel through sea water and the earth’s crust with relatively slow loss of energy but S waves (shear waves) do not propagate at all through fluids. The advantage of OBSs for recording on the seabed rather than using towed arrays to record in the water column is that OBSs can record seabed shear waves directly as vectors using three component geophones, thus providing much useful additional information to inform the interpretation process.

**Figure 2.**
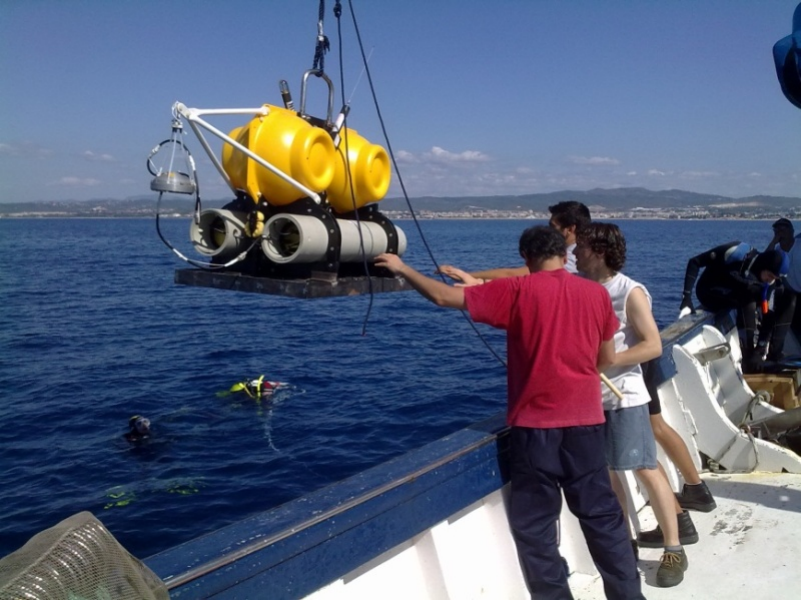
OBS ready for deployment.

Seabed refraction seismology for oceanic exploration has received little attention compared to traditional reflection techniques until recently, and is only now being exploited as a commercial exploration technique. So it is not surprising that related technology has only been developed to a limited extent. As a consequence the hardware equipment necessary for signal acquisition, the ocean bottom seismometer as shown, for example in Figure 2, usually has not been subjected to industrial design discipline. Instruments are often made ‘in house’ by each institute. This makes the test and validation difficult: oceanic exploration takes a long period of time, and experiments simply to test instruments are expensive so very little good intercalibration data exists between different OBSs, and the techniques for obtaining good coupling between sensors and seabed are experimental following a few basic rules like geophone weight, stiff ground, *etc.*

**Figure 3.**
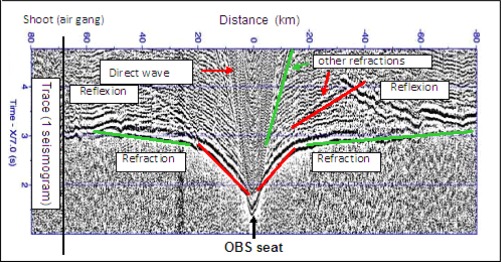
Seismic section from data of a single component, acquired by a single OBS designed by SARTI-UPC in a controlled source seismic experiment. We identify the direct wave, the reflections and refractions of an acoustic wave.

Because of the importance of seismic data to the oil exploration and extraction industries the processing of industrial seismic data has received a great deal of attention, and much development and innovation has taken place. However, most of the industrial data has been acquired using towed hydrophones, and industry has historically had less experience of processing datasets that include multicomponent geophone data, although recently there has been more interest in such data sets because the industry has begun to use seabed recording of seismic data routinely for time lapse monitoring in the vicinity of oil reservoirs. Figure 3 shows a typical raw dataset for a single component presented as a time-distance plot.

In its basic form, processed seismic data gives velocity-depth information for the subsea crust, and shows interfaces between layers and velocity gradients within layers. The physical and chemical properties of the different layers must be deduced by indirect methods [5,6], such as comparison with data from wells drilled into contiguous structures, or from comparison with similar areas. In its most basic form, the interpretation depends upon the identification of the arrival times of wavelets at the recording sensor—these wavelets represent the different phases of seismic signal that are propagated through the seabed, including pressure waves, shear waves and surface waves, with each different ray path through the sea bed giving an additional wavelet arrival. The resolution with which the crustal structure can be determined depends upon the spacing between sensors and also upon the spacing between shots. For a high resolution seismic survey it is necessary to ensure suitable instrument and shot spacing, which is usually determined by modeling the ray paths through a hypothetical model of the survey area to ensure uniform ray coverage [7,8].

**Figure 4.**
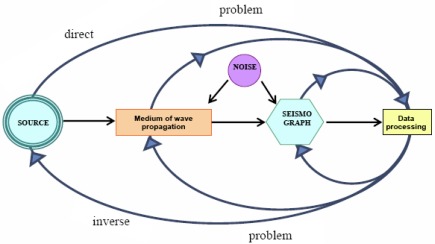
Diagram illustrating seismology as the analysis of complex information system linked to a diversity of specialized and interdisciplinary task of research and application [9].

Figure 4 shows a seismological process diagram as a complicated system and information chain with its many interrelated sub-systems such as the seismic source, wave propagation through the Earth, the masking and distortion of “useful signals” by noise, as well as the influence of the seismic sensors, recorders and processing techniques on the seismogram [9].

As illustrated in Figure 4, the acquisition and processing of controlled source seismic data is a complex process depending upon many steps. Many of these steps are directly connected with experiment planning, data collection planning and instrument and recording systems design, and can be summarized as follows:

1. Choice of suitable survey area to answer the scientific questions.
2. Verify that environmental conditions are suitable.
3. Choice of recording system and seismic source.
4. Design of experiment including instrument and shot spacing to give good ray path coverage.
5. Choice of instrument position to give best chance of good signals.
6. Design of sensor system to give optimum coupling between seabed and sensor.
7. Design of recording system to optimize signal to noise ratio and dynamic range.
8. Provision of precise timing information for data interpretation.
9. Provision of basic data recovery and compilation of industry standard data set.
10. Choice of interpretation methodology.

In this paper we are concerned with items 6, 7 and 8 related to the instrument design, and with the overall mechanical design of the instrument as a suitable delivery vehicle for the sensors and recording system.

**Figure 5.**
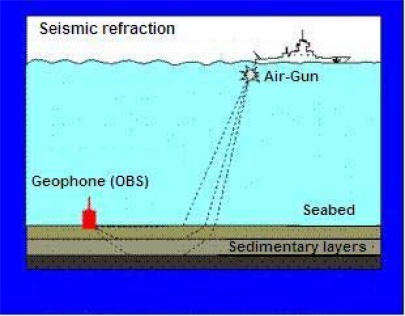
Schematic of a marine seismic refraction (controlled source) survey.

The signal generated by air-guns (Figure 5) may travel many of kilometers through the earth layers before it reaches the sensors, and will therefore be of low amplitude. Careful design of the experiment including the air-gun source size and ranges should ensure that the signal arriving at the sensors is greater than the ambient noise level in the sea. The geophone sensor housing is designed to couple well to the seabed, so that the full seismic ground signal is converted to geophone movement, and the resulting electrical output is maximum. The hydrophone is a piezoelectric sensor that converts pressure signals in the water into high impedance electrical signals, and these are buffered by a high input impedance amplifier. The analogue signal path between sensor and analogue to digital converter is designed so that it does not introduce additional noise; this is aided by having a high sensitivity geophone. Optional pre-amplifier gain can be inserted to ensure that the analogue signal is large compared to the bit size in order not add significant digitization noise into the signal.

The OBS is essentially a delivery and recovery system for the seismic sensors and recording system. The system is designed to work in water depths down to 6,000 meters for durations of up to three months at a time in order to be able to run complex experiments. The OBS is deployed with the geophone suspended on a short electrolytic release that is activated on immersion in sea water, and takes about 2 h to burn through. An hour or so after the OBS has settled on the seabed the electrolytic link releases and the geophone falls under gravity through about 300 mm to land on the seabed. The bottom plate of the sensor housing is designed to displace soft mud sideways from beneath the housing. On completion of the seismic shooting a coded acoustic signal is sent to the OBS and a motor driven release actuator releases the OBS structure from the anchor weight so that it rises due to its buoyancy. The instrument rises a meter or so before the geophone cable becomes taut, so that the OBS has some momentum with which to extract the geophone from the soft mud of the seabed. On reaching the surface the drop in hydrostatic pressure switches on a radio beacon and a xenon flashing light for night recovery. The radio beacon has a range of about 4 miles and is used in conjunction with a direction finding receiver on the recovery vessel.

There are several countries working on refraction seismology during the last decade, but each of them uses their own equipment, which makes comparisons difficult. Table 1 tabulates the comparison.

## 2. The Mechanical Design of the OBS

**Figure 6.**
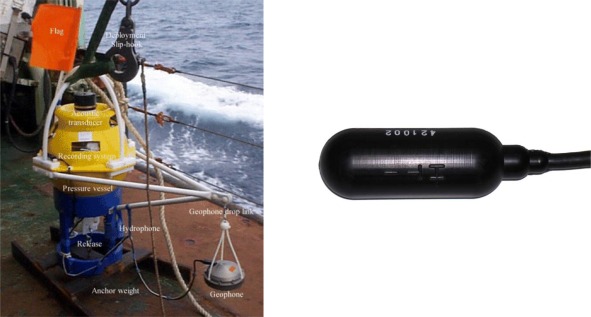
Different parts of the OBS preliminary design using glass sphere. Hydrophone detail from Hytech Company.

In our first design, the miniDOBS, a polyethylene structure protected a single glass sphere (432 mm diameter and 17.7 kg mass in air). The sphere contained the acquisition electronics, data storage, instrument release and the batteries for the electronic system [11] (Figure 6), thus providing both the buoyancy and the housing. In the new UPC-SARTI design the volume available for electronics and batteries has been considerably increased, and the use of glass spheres as instrument housings has been abandoned to make access to electronics and batteries at sea safer and easier. The design uses two sealed 430 mm diameter glass floats for buoyancy, giving a total available buoyancy of 50 kg. By using a high strength aluminum alloy we have been able to provide two instrument/battery housings of 150 mm internal diameter and 850 mm length each with a housing net weight in water of around 5 kg, giving about 20 kg available payload for the tubes (Figure 2). See Table 2 for details of the system weights and buoyancy calculations.

**Figure 7.**
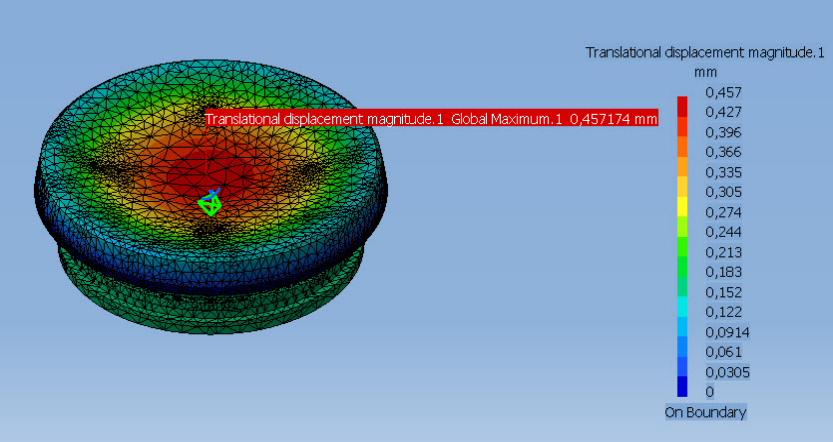
Strength analyses on the cylinder top at 6,000 m of depth.

Buoyancy and drag calculations indicate a rise and fall rate of around 1 m/s, which agrees with initial observations. In Figure 2 is shown the UPC-SARTI’s OBS mechanical system designed by Carrack Measurement Technology. The pressure cylinders were designed using Finite Element Modeling (Unigraphics 2.0, by Siemens PLM Software) to give a safety factor of 1.4 at 6,000 m (Figure 7). The tubes were bored from solid bar and then heat treated T6 to give maximum strength before finish machining (T6 Heat Treatment is a specific heat treatment process which may be applied to aluminum/copper/silicon alloys to increase the strength of the alloy by as much as 30). After hard (sulfuric acid) anodizing, hydraulic tests were carried out at the Pressure Test facility at the National Oceanography Center in Southampton (UK) to the equivalent of 6,000 m depth in seawater, including a soak test at full pressure for 24 h. No leaks were detected. See Table 3 for the tube specifications.

### 2.1. Geophone

**Figure 8.**
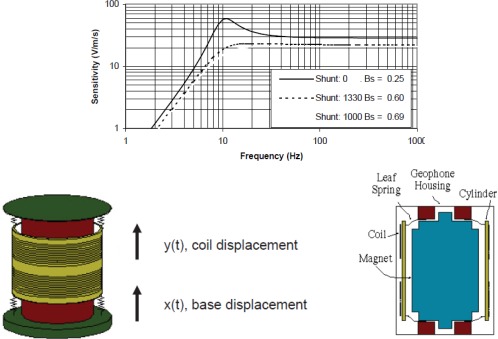
Characteristic curve sensitivity/frequency of the SM-6 sensor (28.8 V/m/s is a maximum sensitivity at 8 Hz from Input-Output Company, Stafford, TX, USA). An electromagnetic sensor diagram.

**Figure 9.**
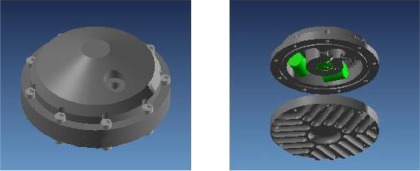
Geophone structure and transversal section where the space for the sensors can be seen. The contours in the base are to improve coupling with seabed mud. Based on a design by Carrack Measurement Technology.

Main sensors in the OBS are the hydrophone, which is sensitive to pressure fluctuations within the near seabed water column, and the geophone that responds to movement of the seabed itself. The geophone has three perpendicular components; it has been designed from small, robust and high sensitivity magnetic velocity sensors (SM-6 with 28.8 V/m/s from Input-Output Inc. Stafford, TX, USA. and GS-11 with 85 V/m/s from OYO GeoSpace Technologies, Houston, Texas, USA). This sensor contains a magnetized seismic mass surrounded by a coil—movement of the mass causes the magnetic field lines to cut the coil, which induces an electric current through the coil (Figure 8). The signal is amplified and is proportional to the velocity of movement of the surface in the direction of the sensor axis where the geophone is placed. The geophone housing contains the three geophones. It has been designed using 3-D finite element software (COSMOS) to work down to a seawater depth of 6,000 m, (Figure 9). The design is based on the use of aluminum alloy 3005-H18.

The performance of the geophone system depends upon several different parts of the system, with the relative importance of each depending upon the details of the application. The factors that govern the overall performance of the system are; the fidelity with which the geophone housing follows the ground motion, the signal to noise ratio and linearity of the geophone sensors, the signal to noise ratio and dynamic range of the amplifier stages and the resolution, dynamic range and noise performance of the analogue to digital converter stage. Fidelity also depends upon the timing and converter clock. Once the signal is in digital form, its quality should be unchanged by the system.

The most difficult part of the design of any geophone is to ensure that the signals recorded accurately represent the ground motion in all components. This requirement is complicated by the nature of the seabed in most deep water locations where sedimentation occurs more or less uninterrupted. In these locations the seabed has a density gradient near the surface because the sediment only compacts slowly under its own weight, and even when compacted the fine particle material is surprisingly elastic. The upper layers—probably the upper few cm—can be very soft, often more liquid than solid. To get the best coupling using a gravity deployed geophone sitting on top of an elastic seabed with layers of very soft mud, the geophone must displace the softest material in order to rest on a more solid seabed. This requires an optimized base loading factor. The design of the housing must also be optimized so that seabed motions are followed accurately, without generating spurious modes, for instance due to rotations of the geophone housing about horizontal axes. Assuming that the geophone housing follows the ground motion accurately, signal quality depends upon obtaining a good signal to noise ratio throughout the analogue signal path.

Moving coil geophones are designed to work above their resonant frequency, which depends upon the moving mass and the spring constant of the restraining suspension; they are not suitable for recording signals below a few Hz, and thus are not used for earthquake seismology where the useful signals are typically below 1 Hz. In the region above resonance the sensor acts as a velocity sensor, and in controlled source seismic applications this gives a fairly flat signal spectrum across the signal frequency range, thus making best use of the dynamic range and signal to noise ratio of the analogue signal path. The inherent signal to noise ratio and dynamic range of small moving coil geophones makes them suitable for use in controlled source OBSs, and the small level of harmonic distortion they produce due to non-linearities in the suspension is generally not significant.

Depending upon the geophone sensitivity and the analogue to digital converter bit size and saturation input level it may be necessary to introduce an analogue signal amplification stage for optimize signal to noise ratio. It is necessary to optimize the design of the signal path for dynamic range and signal to noise ratio to preserve data integrity.

It is very important to know the S/N ratio in the sensors used in seismic refraction, since the signal obtained is very weak in front of a noisily environment. In order to adjust the gain of the amplifiers and characterize the frequency response of the set sensor and amplifier, it has been developed a model of a magnetic sensor from generic models of sensors [12]. Equation (1) expresses the transfer function H_1_(s) of the magnetic geophone according to the output current I(s) respect to removal in one axis Z(s):

(1) $$H_{1}(s)=\frac{I(s)}{Z(s)}=\frac{K\cdot s^{3}}{s^{3}+\left[\frac{R}{L}+\frac{\tau }{m}\right]s^{2}+\left[\frac{S+\tau \cdot R/L+K^{2}/L}{m}\right]s+\frac{\text{SR}}{\text{mL}}}$$

where K = 2πnrH in Henry is the constant sensor where n is the number of turns of the coil and r the radius, R is the resistance of the coil, L inductance of the coil, S is the elastic constant of the spring, m the inertial mass and τ mechanical damping factor.

When L ≈ 0 in Equation (1), the s^3^ term in the denominator can be neglected due to the range of frequencies considered. If in addition, we transform the current I(s) to tension by means of an external resistance Rs, (1) can be approximated by:

(2) $$H_{1}(s)=\frac{R_{S}I(s)}{Z(s)}=\text{sL}\frac{K\cdot R_{S}\cdot s^{2}/R}{s^{2}+s\left[\frac{\tau }{m}+\frac{K^{2}}{\text{mR}}\right]+\frac{S}{m}}$$

Hence, the speed is expressed as a derivative of the displacement, the Laplace transform became S(s) = s. Z (s) yielding the transfer function:

(3) $$H(s)=\frac{V(s)}{S(s)}=\frac{K\cdot R_{S}\cdot s^{2}/R}{s^{2}+s\left[\frac{\tau }{m}+\frac{K^{2}}{\text{mR}}\right]+\frac{S}{m}}$$

The Function (3) gives the voltage output V(s) according to the input speed S(s). Its units are V/m/s. This function is analogous to the transfer function of a high-pass H(s) second order filter with natural frequency ϖ_o_, damping factor ξ, and gain H_o_ of the form:

(4) $$H(s)=\frac{H_{o}\cdot s^{2}}{s^{2}+2\xi \varpi_{o}s+\varpi_{o}^{2}}$$

**Figure 10.**
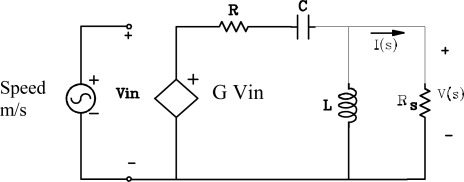
Model of the geophone with passive elements.

This identification implements a model to approach the frequency response of the sensor via electric circuits. A first model is based on passive elements [13], including a controlled source to obtain the needed gain. This model is shown in Figure 10.

The transfer function for the circuit in Figure 10 is:

(5) $$H(S)=\frac{V(s)}{S(s)}=\frac{G\cdot \frac{R_{S}}{R_{S}+R}\cdot s^{2}}{s^{2}+\left[\frac{R_{S}\cdot R}{\left(R_{S}+R\right)L}+\frac{1}{\left(R_{S}+R\right)C}\right]\cdot s+\left[\frac{R_{S}}{R_{S}+R}\right]\frac{1}{\mathrm{CL}}}$$

**Figure 11.**
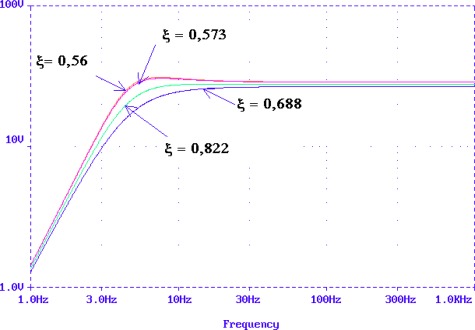
Frequency responses in dB of the model RCL.

The Input-Output sensors specifications provided by the manufacturer gives R = 375 Ω, the constant generator G = 28,8 V/m/s and natural frequency, f_o_ = 4.5 Hz. From these data it is possible to obtain values for the capacitor, coil and resistance Rs in the model and calculate the appropriate damping factors. In the transfer function we can identify the parameters R, C and L. The PSpice simulation of the RCL model is shown in Figure 11 and the results obtained agree with Input-Output sensors manufacturer specifications for the SM-6 geophone (Figure 8)

Oceanographic campaigns are very expensive as to spoil them with poor performance equipment. Therefore, the specifications and performance of designed equipment must be verified with reliable test processes. In order to prepare measure equipment with wanted specification and performances in range of frequency, S/N ratio and good coupling seabed, we need to simulate a proposed model, which is subsequently validated in the lab and in the final test. To characterize the underwater geophone we need a precise model to obtain the correct simulated answer.

**Figure 12.**
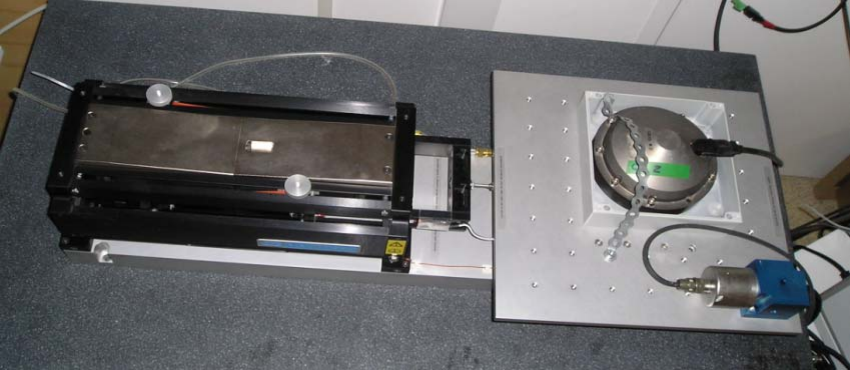
Shaker table with geophone and accelerometer.

The model of a geophone has been compared with the experimental results at the laboratory. With this aim the geophone structure built using Aluminum_6082-T6 including SM-6 or GS-11 magnetic accelerometer has been tested using a vibration transducer calibrator (Beran 455 from Beran Instruments Ltd. Devon. United Kingdom) and a shaker table (APS Model 129 from APS Dynamics, Inc., San Juan Capistrano, CA, USA), see Figure 12.

**Figure 13.**
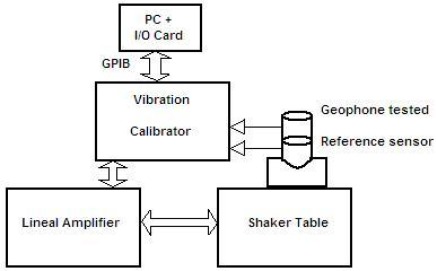
Block diagram of the measurement system.

An application developed using LabVIEW language programming from National Instruments (Austin, TX, USA) registers the measurements and stores them in a data file via GPIB Bus with an acquisition card. Figure 13 shows the block diagram of the measurement system with the communication between the PC, the vibration calibrator, and the shaker table in order to obtain the measure of the geophone output sensitivity in V/m/s.

**Figure 14.**
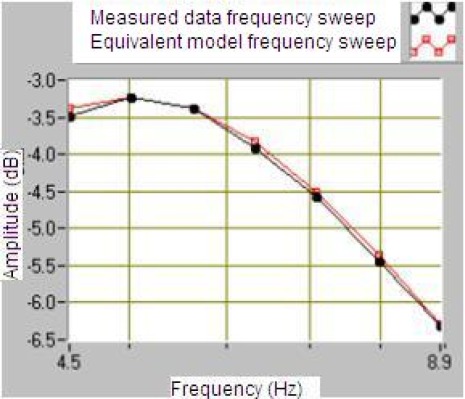
Comparative with data measured and calculated of the geophone SM-6 sensitivity in dB through the frequency.

**Figure 15.**
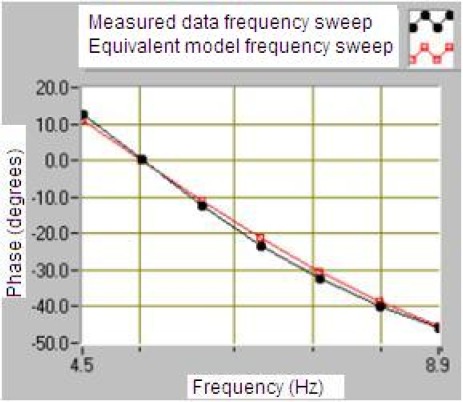
Comparative with data measured and calculated of the geophone SM-6 sensitivity in degrees through the frequency.

The LabVIEW program allows the communication with a vibration calibrator and prepares them to measure the sensitivity of the geophone. The results of this program are the plots of frequency response shown in Figures 14 and 15 for a module and phase behavior sensitivity (performance). From this plots it is possible to obtain the natural frequency ω_o_, the damping ξ_o_ and the rest of the parameters of the magnetic geophone and the final test is to calculate the results of the mathematical model and compare them to a measured data.

The results in Figures 14 and 15 prove that the proposed velocity model is correct because its error is lower than 6% at frequency before the ω_n_ and lower than 1% after them in module frequency response. As far as the phase performance of the model proposed the error is lower than 4 degrees. This allows validating the velocity of the geophone model and the parameters given by the manufacturer. This method allows testing several models of the geophone in order to characterize them better.

The leveling problem when the geophones are installed on the seabed would be resold including a tilt meter sensor in the geophone structure, but in our design it has chosen a solution based in the register data of the triaxial geophone. A calibration procedure in the range 8 Hz until 64 Hz has been done for GS-11D sensor and the sensitivity results are shown in Table 4.

The geophone was put on the shaker 60° respect axis 2 (Figure 12), and the Table 5 shows the test results. The compared values show is possible to determine the velocity direction of the propagation in the plane XY and their uncertainties.

## 3. Electronic Design

**Figure 16.**
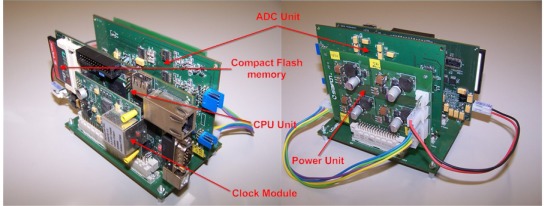
Packaging of the OBS electronic units.

The electronic acquisition system consists of four main blocks: analog-to-digital conversion module, micro-controller and data storage module, power regulation module and time base module. A lithium-ion battery pack is built and used as the main power supply. Figure 16 shows the packaging of the acquisition system implemented:

The micro-controller and storage module is based on a MCF54455 microcontroller and a 4 GB Compact Flash memory card for data storage. This module is in charge of configuration of the ADC module selecting the sampling rate and low power operations. The acquisition software designed acquires data continuously from input channels through a QSPI (Queued Serial Peripheral Interface) bus, performs time stamping by using the integrated RTC (Real Time Clock) and stores the data in the Compact Flash memory card. Furthermore, this module carries out the following functions:

- Default configuration of the system
- Time synchronization with a GPS signal
- Clock drift calculation
- Low power sleep.

### 3.1. Analog-to-Digital Conversion Module

This section presents the design and implementation of a high resolution, low noise acquisition system aimed for marine seismology. To find the specifications of the system built before it is used in real environmental conditions, a series of characterization tests based on the IEEE Std-1057 and IEEE Std-1241 standards are implemented. In order to obtain realistic results in these tests, external noise coupling to the system is minimized. Keeping in mind the final application, parameters as Signal-to-Noise Ratio, Dynamic range, Effective Number of Bits (ENOB) and ADC clock jitter are calculated (Table 6).

In order to reduce the noise coupling from power regulation module to the ADC module as well as maximizing the autonomy of the instrument, MAX1653 switched converters with output noise control are used. This regulator reduces interference due to switching noise by ensuring a constant switching frequency regardless of the load and line conditions, thus concentrating the emissions at a known frequency outside the system bandwidth.

**Figure 17.**
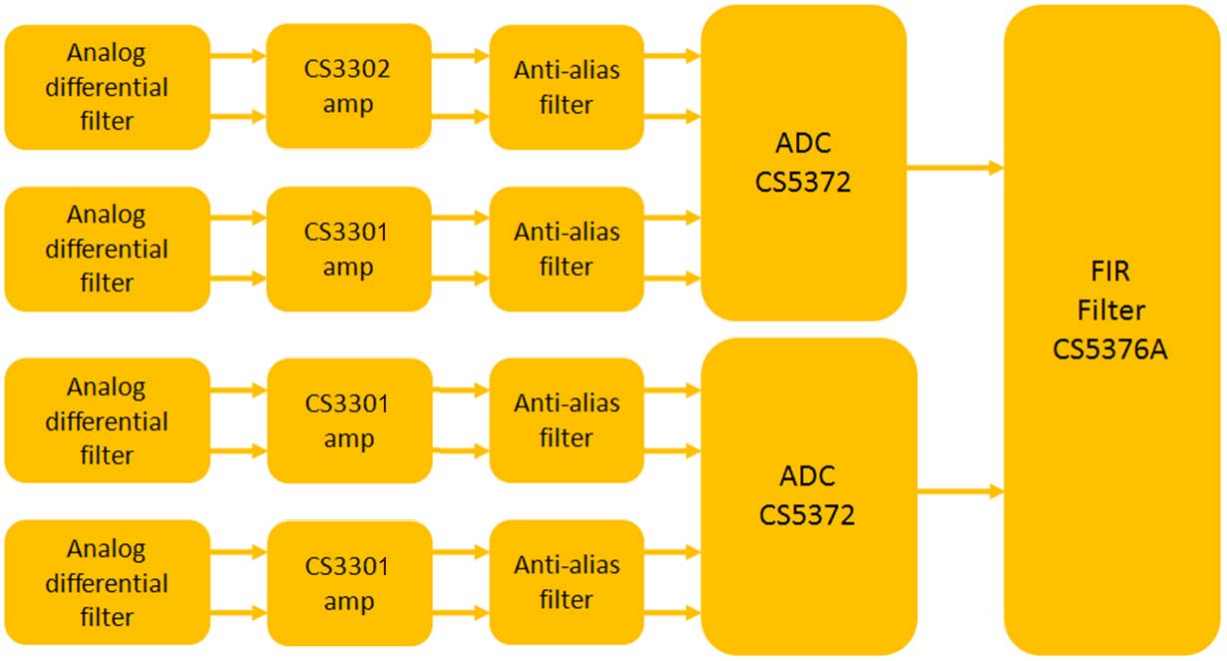
Block diagram of the ADC module.

The noise level of the Analog-to-Digital conversion module defines the final resolution of the overall system. Special care must be taken in designing this module in order to have high quality data. The ADC module is based on a 4 channel 24 bits CS5372 ADCs oversampling at 512 kHz and a CS7276A digital filter for data decimation and interfacing with the microcontroller module. The CS5372 are two channel high dynamic range, fourth order Δ-Σ modulators which provides a dynamic range of 130 dB@103 Hz bandwidth and a Total Harmonic Distortion (THD) of −118 dB. Figure 17 shows the block diagram of the ADC module.

The acquisition systems aimed for controlled source seismic surveys require a low noise level, thus maximizing the ENOB as well as dynamic range. Electromagnetic interference (EMI) together with channel crosstalk is a common cause of signal degradation in high resolution acquisition systems. A poor design of the ADC module results in a high noise level and therefore a low signal-to-noise ratio.

The following considerations were taken into account in the design of the ADC module in order to obtain the required noise performance:

The input stage of the ADC module is based on the CS3301/02 low noise fully differential amplifiers with a noise density of 8.5 V/√Hz over the bandwidth. Analog fully differential signaling presents many advantages in low level signal acquisition. First the signal is protected from the noise coupling paths which are reference and power supply and second, by definition, the signal amplitude is double of that in single-ended signals. Differential signals also improve EMI immunity which directly depends on the return current loop areas in the PCB. In order to reduce EMI within the analog stage of the PCB, the areas formed by the currents flowing through tracks and the return currents that flow under the tracks have to be minimized. In differential signals, this is achieved easily as the return current related to the negative terminal has the same magnitude and opposite sign of the one that appears on the positive terminal and therefore the net current cancels out. This is only true if the positive and negative terminals have the same length. Therefore in the PCB design, differential traces have to be parallel. By minimizing the distance between parallel traces, current loop areas are also minimized resulting in reduced EMI [14]. Each device supply pin includes a 100 nF capacitor for high frequency noise rejection, and a 10 μF capacitor for low frequency noise rejection. Both capacitors are placed as close as possible to the supply pin with the lower capacitor placed closer to the pin to reject the noise generated by the trace itself. Furthermore, special care has been taken in choosing the capacitor technology for analog filtering, anti-alias RC network and the voltage reference of the ADCs, specified by the ADC manufacturer.

We have designed and built a data acquisition and storage system optimized to acquire the controlled source seismic signals. Also the characterization tools based on international standards have allowed us to qualify the system performance. Table 6 summarizes the results of the system performance for a sampling rate of 250 samples per second.

### 3.2. Microprocessor Module and Data Storage

**Figure 18.**
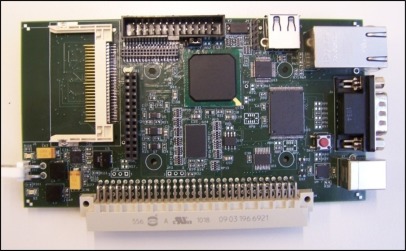
MCF54455 processor module.

The main processing unit design is based on a MCF54455 microcontroller. This highly-integrated 32-bit microprocessor, with its internal peripherals, is the main core of the seismometer processing unit board presented in Figure 18.

The system is designed to have these features:

Power circuit:
  - MC34717EP integrate circuit, used to provide the 1.8-V and 1.5 V from the input voltage of 3.3 V
Memory:
  - 16 Mb Flash memory
  - MT47H32M16 SDRAM.
  - CY62167EV30 2 MB SRAM memory directly connected to the Flexbus
Interface:
  - RS232 serial port for user interface.
  - SPI port connected with the ADC board.
  - ATA port connected with the Compact Flash (CF) memory.

**Figure 19.**
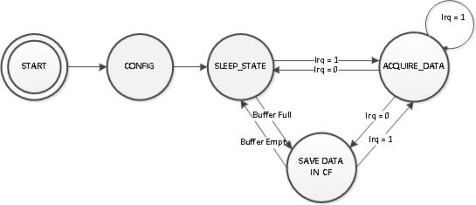
OBS state machine.

The acquisition software running on MCF54455 microcontroller, acquires data continuously from ADC module, performs time stamping and stores the data in the Compact Flash memory card as presented in the state machine depicted in Figure 19.

### 3.3. Power Supply Module

**Figure 20.**
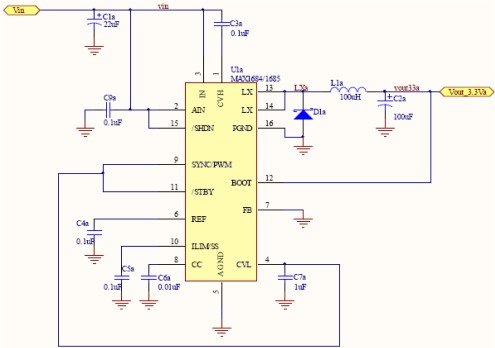
Power circuit schema of the 3.3 V for microcontroller board.

In addition to the electronics unit, a power unit has been designed to provide all necessary system voltages. Therefore, to feed the seismometer system, you need a 3.3 V supply to power the microprocessor board and three voltages of 3.3 V, 2.5 V and −2.5 V to power the acquisition board. The two power supply voltages of 3.3 V are generated independently in order to avoid interference between them. This is mainly because the acquisition board requires stable power supply with low noise characteristics. The power supply corresponds to a set of batteries that provides a voltage of approximately 3.6 V. The four power circuits, follows the same schema as in Figure 20. Selecting the right components we generate the two 3.3 V, 2.5 V and −2.5 V.

### 3.4. Time Base Module and Clock Stability

In controlled source marine seismology, the quality of the ocean bottom model obtained after signal processing is related directly to the timing of the acquired data. As the equipment has no access to a GPS signal for time synchronization during the experiment, a single precise crystal is used to generate all the signals necessary for data acquisition. The time base module uses as the main time reference a Seascan SISMTB version 4.0 clock which has been the device under test with a specified temperature stability of 2 × 10^−8^. This module is a Temperature Compensated Crystal Oscillator (TCXO) [15,16] which uses a microprocessor based digital temperature compensation scheme to synthesize a compensated reference frequency of 125Hz from the output of a low aging and free running quartz oscillator. Any marine seismic application needs a great stability of the timing unit with temperature as there is no access to the instrument for time synchronization. Therefore all the necessary signals for the datalogger to function correctly, have to be generated from a single crystal and take into account the stability requirement.

**Figure 21.**
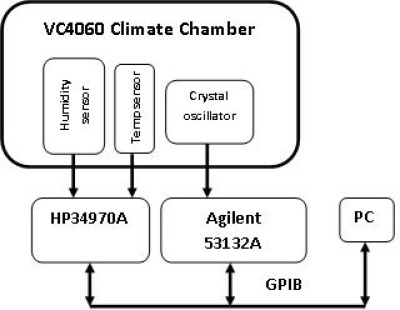
Measurement system block diagram.

In an experiment, we placed the crystal oscillator inside a VC4060 environmental chamber where temperature is controlled. In order to measure the temperature close to the Seascan module, a temperature sensor is placed beside it and 4-wire measurements of the sensor are carried out. The available version of the VC4060 chamber does not allow humidity control below 10 °C inside the chamber. A humidity sensor is used to measure the humidity near the crystal. A HP34970A datalogger is used to measure the temperature and an Agilent 53132A universal counter with a temperature stability of 2.5 × 10^−9^ was used to measure the Seascan output frequency (125Hz). In order to obtain an improved resolution, frequency is measured within a time gate of 1 s. The overall measurement system is controlled by a PC through a GPIB bus, where software in LabVIEW 8.5 takes measurements every 20 seconds. Figure 21 shows the measurement system in the lab:

In order to obtain the *Frequency-Temperature (F-T)* characteristics [17,18] of the Seascan module in the OBS operation temperatures, the temperature is changed between 0 °C and 25 °C during a few hours. During the tests, the temperature profile is configured to simulate the OBS real operation (laboratory, on board, in the sea water):

1. From 25 °C to 0 °C in 8 hours;
2. At 0 °C constant for 8 hours;
3. From 0 °C to 25 °C in 8 hours;
4. At 25 °C constant for 8 hours.

This profile was cycled twice to show data consistency. The time base module stability is calculated as:

(6) $$\mathrm{Stability}=\frac{f_{i}-f_{\mathrm{nom}}}{f_{\mathrm{nom}}}\;$$

In order to measure the time drift of an OBS in a controlled source seismic experiment, before deployment the instrument clock is synchronized with a GPS signal. After the OBS recovery, the instrument clock is compared to the same GPS signal and the time drift is calculated as the time difference. In the data processing stage, OBS data are corrected by applying a linear time correction.

In order to calculate the time drift of the Seascan module in the lab, a long period test was carried out using the measurement system shown in Figure 21. In this test, the temperature inside the climate chamber was set to 0 °C. Two Seascan crystals (SN-968 and SN-969) were used and the duration of the test was about 40 days as an controlled source seismic experiment at ocean takes about three to four weeks. This test was repeated twice with one module (SN-968) to verify consistency of the results.

The time drift was calculated from the frequency measurements as follows:

(7) $$\mathrm{Timedrift}=\frac{\sum_{i=1}^{n}\left|\Delta f_{i}\right|}{f_{\mathrm{nom}}}T_{\mathrm{samp}}$$

where *T_samp_* is the measurement interval which is 20s in this test, and:

(8) $$\Delta f_{i}=f_{i}-f_{\mathrm{nom}}$$

where *f_i_* is the frequency measurements and *f_nom_* is the nominal frequency.

**Figure 22.**
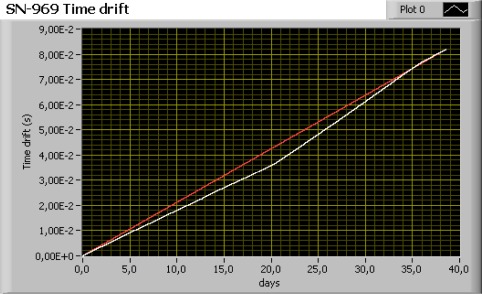
Time drift of the Seascan module (SN-968). The white line is the time drift of the crystal measured. The red line is the time drift correction of the OBS data.

In Figure 22, the results of this test show that the residual time error after correction assuming linear drift ranges between 2 ms and 6 ms for a recording period of 40 days. This value is similar to the sampling rate used in controlled source seismic experiments, which means that the time correction error will barely affect the seismic modeling results.

Normally after the OBS is recovered from a measuring campaign, the crystal clock drift can be compensated if it has been measured previously and it has a linear behavior. If the behavior is not linear, the only solution is to minimize the clock drift using Oven-Controlled Crystal Oscillator (OCXO) crystals, that are much more stable but with higher energy consumption. Therefore the OCXO crystal power consumption has a great impact on the OBS autonomy.

In the following section a cabled OBS system is presented as an improved solution for time synchronization, capable of sub-microseconds accuracy.

## 4. Cabled OBS and Clock Synchronization

For studying and understanding the generation processes of earthquakes near the coast, it is important to observe seismic activities on the sea floor just above these seismogenic zones. Cabled Ocean Bottom Seismometers are the best solution to address these problems. For this a cabled version of the OBS described above has been developed to be installed in the OBSEA observatory. OBSEA is a test-site cabled seafloor observatory 4 km offshore Vilanova i la Geltrú coast (Barcelona, Spain) located in a fishing protected area, and interconnected to the coast by an energy and communications mixed cable [19]. The main advantage of having a cabled observatory is to be able to provide power supply to the scientific instruments and to have a high bandwidth communication link. In this way, continuous real-time data is available. The proposed solution is the implementation of an optical Ethernet network that transmits continuously data from marine sensors connected to the observatory.

An important requirement to have an accurate location of the earthquake is to provide precise time-stamping of seismic data with GPS reference. Without the possibility to have GPS signal in underwater environment, the only way to transmit such a signal was to dedicate a single wavelength to timing. Commercial products are now becoming available that support IEEE 1588 Standard for a Precision Clock Synchronization Protocol for Networked Measurement and Control Systems [20]. IEEE 1588 should allow sub-microsecond timing accuracy in a pulse per second signal at the seabed junction boxes. Precision time protocol (PTP) equipment will be installed on the shore station working as a Grand Master (GM) synchronized GPS. Extraction of the timing is the responsibility of the seismic instruments, whether the signal is pulse per second or simply the time stamping of data. The PTP can also be available to other instrument that needs sub-microsecond timing accuracy.

**Figure 23.**
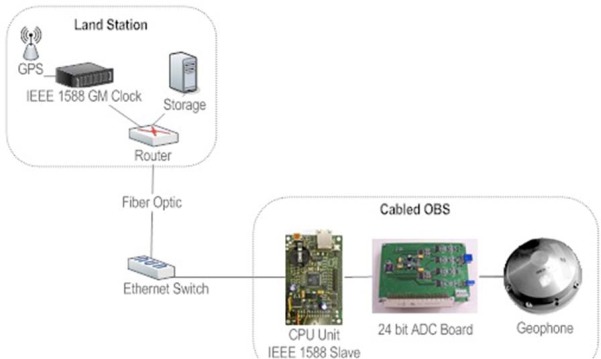
Luminary LM3S9B96 Broadband Seismometer with IEEE 1588 synchronization.

The proposed cabled seismometer is designed based on the autonomous OBS from UPC and adapted to Ethernet communication. The controller used in this system is a Stellaris^®^ Luminary (Texas Instruments) LM3S9B96 with IEEE 1588 PTP hardware support (Figure 23).

In this system, the real time clock of the LM3S9B96 board synchronized with GM Clock is used to timestamp the seismic data. The PPS signal from the LM3S9B96 is used with a PLL to generate the sampling frequency for the ADC converter.

**Figure 24.**
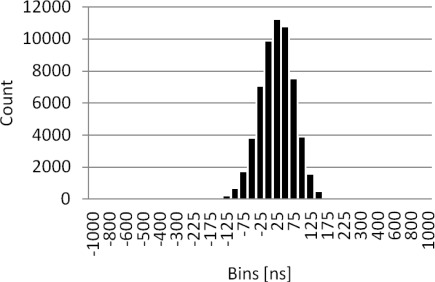
PPS signals offset histogram with PTP IEEE1588 using COTS Ethernet Switch with 100 Mbits traffic and VLAN setup.

To evaluate the precision achieved by the slave clock, a histogram of the offset between the master and slave clock pulses was generated. The test was done simulating the OBSEA network with the seismometer connected to the junction box Ethernet switch. The clocks offset measurement has been done for 100 Mbits data traffic and VLAN setup was used to separate the IEEE 1,588 capable instruments and master clock from generated traffic. The results after more than 48 h of tests are presented in Figure 24 showing an average offset between pulses of 51 ns. The peak to peak jitter is 5 μs equivalent to ±2.5 μs accuracy.

## 5. Conclusions

In this paper we present the design and construction of a marine seismograph following the scheme of a measuring instrument as a chain of blocks from the sensor, that receives the information signals to be acquired, to registration and display of the information. In the first part it has been presented the sensor, in our case the geophone. Further all the electronics of the acquisition system and computer control unit has been presented, with an emphasis on clock synchronization. Throughout the construction of the marine seismometer prototype, calibration standards have been used to allow us to know the uncertainties in the measurements.

It has been designed and characterized a high sensitivity geophone for marine applications. This device, together with a four channels acquisition system, constitutes a small marine seismometer. This feature makes it extremely useful since it can be transported and manipulated in a small ship, and an oceanographic vessel is not needed. It can be used in civil engineering, submarine surveying, and mine prospecting, and also in taking high-resolution views of the cortical structure [21].

The main objective in the design of the geophone is to obtain maximum sensitivity, trying to make the geophone work near the resonant frequency. To make the coupling to the marine bottom easy and get the maximum sensitivity, the container has a certain profile at its base. Although some rocks can make the coupling more difficult, usually the ocean bottom is soft enough to get a good horizontal coupling. On the other hand, we have to work with signal amplifiers with their maximum gain avoiding the saturation zone. It is very important to know the S/N ratio in the sensors used in seismic refraction, since the signal obtained is very weak in front of a noisy ambient. In order to adjust the gain of the amplifiers and characterize the frequency response of the set sensor and amplifier, we have developed a model of a magnetic sensor from generic sensor models and we can distinguish the Input-output magnetic sensor which is the reference of a designed geophone. Since this model has been implemented we have built a simulator of frequency behavior of the geophone, in order to characterize the rest of the electronic measurement system without the physical presence of the geophone, and to know how the housing changes affect the geophone behavior [10].

In the last decade, many marine research institutes have designed and implemented their own acquisition systems for their Ocean Bottom Seismometers (OBS). However, at present there is a lack of standard procedures for characterization of these systems. Every marine institute provides specifications of their acquisition system but they do not give any indication of how they have been calculated. In order to find the performance of the data logger built, a series of tests based on the “IEEE Std-1057 Standard for Digitizing Waveform Recorders” and “IEEE Std-1241 Standard for Terminology and Test Methods for Analog-to-Digital Converters” are implemented in LabVIEW [11]. Taking into account that the system under test has a 24 bits resolution and a noise analysis is necessary, low noise instruments have to be used in order to calculate the parameter correctly. In this case, an ultra-low noise and distortion Stanford Research System (SRS) DS360 function generator is used to generate the input signals. The DS360 function generator presents a −110 dB Total Harmonic Distortion (THD) in 5 kHz bandwidth with a minimum 20 μVpp differential amplitude allowing characterization of the system by using low input amplitudes as in real conditions. Another common source of noise coupled to the system is the power supply. Power supplies are often not filtered and not suitable for low noise systems. The implemented tests described in previous section are used to find the specifications of the acquisition system before carrying out a controlled source seismic experiment. Noise parameters are calculated by using a 20 μVpp differential input sine wave minimizing the noise coupled from the signal generator to the system. This constitutes a realistic case as in controlled seismology the signal level detected by the instrument is very low. The specifications of the data logger show that the dynamic range of all the channels are about 129 dB, slightly below the ADC dynamic range at the same sampling rate (130 dB@103 Hz), while the resolution of the system is about 21.4 bits due to noise. Random noise is about 1.1 LSBs which corresponds to about 320 nV. A channel crosstalk of −147 dB shows a good PCB design and EMI immunity.

The design and test of an acquisition system built for marine seismology has been explained in this paper. Low amplitude signals have to be used in order to minimize the noise coupled from the generator to the system. Comparing the specifications given in previous section with the similar systems built for OBSs, we can conclude that the noise level is low enough and having and autonomy of two months (Table 1), this data logger can be used for mid-term marine seismology.

In this paper, the stability and time drift of a time base module (Seascan) used in most Ocean Bottom Seismometers (OBS) has been investigated. In order to find the *f-T* characteristic of the crystal, temperature was set to cover the OBS operation temperature at sea. The results of the stability test show that the stability degrades around 21.5 °C. This temperature is recognized as the crystal turnover temperature. Furthermore, a long period test was carried out to find the time drift of the OBS clock in real environmental conditions. The results of this test show that the residual time error is around 2–6 ms for a 40 days recording period, so it will barely affect the seismic modeling results in controlled seismic experiments. However, the residual time error could potentially be larger than 50 ms for recording periods of over one year, having a significant effect in the estimation of hypo-central earthquake locations. To obtain a better clock stability, the use of OCXO (oven-controlled crystal oscillator) crystals has been studied [22]. The OCXO and TCXO (temperature compensated crystal oscillator) crystal frequencies show a temperature stability of 49 ppb and 71 ppb, respectively. OCXO presents a power consumption of 1.5 W when the temperature is −2 °C constant while TCXO’s power consumption is 55 mW at the same temperature. The difference in the power consumption of tested crystals is due to the temperature compensation techniques applied. While a TCXO integrates a microcontroller with on-chip calibration data, an OCXO places the crystal inside an oven where the temperature is kept constant by drawing more current. The tests show that the use of OCXO crystals as the time base of the overall system would set a major limit on the instrument autonomy even though presenting a better temperature stability than a TCXO crystal.

To obtain a good clock syncronization and to have large series of real-time data, cabled seismometres are the best solution to address these problems. In this work a cable seismometer has been developed capable of sincronizing his clock through IEEE 1588 standard with an accuracy of less than 1 microsecond.

## Figures and Tables

**Table 1.** Comparative of present Ocean Bottom Seismometers.

	**Woods Hole-D2**	**SIO L-Cheapo**	**JAMS-TEC OBS**	**GEOMAR OBS**	**GEOMAR K/MT 562**	**IRD-UTIG**	**Hippo campe**	**Micr OBS**	**Mini DOBS Carrack**	**UPC SARTI**

**Structure**	2 glass sphere	pressure tube	1 Glass sphere	pressure tube	pressure tube	Glass sphere	2 glass sphere	1 glass sphere	1 glass sphere	pressure tube
**Geophone frequency (Hz)**	L-28 4.5 Vertical-Out	L-22 2 Vertical-Out	3 axis 4.5 In	3 axis 4.5 In	3 axis 4.5 Out	3 axis 4.5 In	3 axis 0.03 Out	3 axis 10 In	3 axis 4.5 Out	3 axis 4.5 Out
**Data logger channels**	2 24 bits	2 24 bits	4 16 bits	4 24 bits	4 24 bits	4 16 bits	4 24 bits	4 24bits	4 24 bits	4 24 bits
**Store**	Hard disc 9 GB	Hard disc 9 GB	Hard disc 20 GB	Hard disc 8 GB	Hard disc 8 GB	Hard disc 2.1 GB	Hard disc 20 GB	Hard disc 1 GB	Hard disc 2.1 GB	Compact Flash 16 GB
**Clock (drift)**	TCXO 3.5 × 10^−8^	TCXO 3.5 × 10^−8^	-	TCXO 5 × 10^−8^	TCXO 5 × 10^−8^	TCXO 3.5 × 10^−8^	TCXO 2 × 10^−8^	TCXO 10^−7^	TCXO 71 × 10^−9^	TCXO 5 × 10^−7^
**Release**	electrolytic	electrolytic	electrolytic	mechanic	mechanic	electrolytic	mechanic	electrolytic	electrolytic	mechanic
**Consume**	1W	540 mW	-	1.8 W	1.5W	-	500 mW	-	-	1W
**Autonomy**	2 months	4 months	20 days	14 days	1 month	2 months	6 months	10 days	14 days	2 months
**Weight**	73 kg	128 kg	97 kg	165 kg	255 kg	85 kg	-	10 kg	70 kg	70 kg
**Height/width/length (m)**	1.1/0.56	0.9/0.6/0.9	0.6/1/1.2	2/1.2	0.6/1/1.2	0.6/1/1	0.6/0.5/1	0.4/0.4/0.4	1.1/0.5	1.1/0.5

**Table 2.** Buoyancy calculations for new OBS.

**Double tube - 2 spheres**						

	wt air	DISP lt	wt water	Qty	kg air	kg water
glass sphere	17.2		−26.0	2.0	34.4	−52.0
aluminum tube	26.38	23.5	2.42	2.0	52.8	4.8
Tube payload (max)	20.0	0.0	20.0	1.0	20.0	20.0
release actuator	2.6	1.5	1.1	1.0	2.6	1.1
release transponder	3.0	1.0	2.0	1.0	3.0	2.0
flashing light	1.7	0.7	1.0	1.0	1.7	1.0
radio beacon	1.6	1.0	0.7	1.0	1.6	0.7
Cables	1.0	0.6	0.4		0.0	0.0
Plastic frame sides	9.0	9.6	−0.6	2.0	18.0	−1.2
Geophone	2.2	1.0	1.5	1.0	2.2	1.5
geophone arm *etc.*	2.0	1.5	0.5	1.0	2.0	0.5

**Rising—nett kg**					**138.2**	−**21.6**

anchor weight	80.0	11.0	69.0	1.0	80.0	69.0

**Sinking—nett kg**					**218.2**	**47.4**

**Table 3.** Pressure tube specifications.

Depth of failure-elastic.	(m)	16,643
Depth of failure-stress.	(m)	7,711
Failure occurs at.	(m)	7,711
Az		25.06
Displacement per meter	(kg)	27.39
Total displacement	(kg)	23.96
Wt in air	(kg)	26.38
Wt in water	(kg)	2.42
**Safe Working depth**	**(m)**	**6,169**

**Table 4.** GS-11D Sensitivity.

	**Sensitivity (V/m·s^−1^)**	U* (V/m·s−1) * The uncertainty
1 ó X	85.78	2.52
2 ó Y	82.79	3.47

**Table 5.** Determination of orientation.

**Sweep**	**Frequency (Hz)**	**Axis 1**	**Axis 2**	**Velocity module (mm/s)**		**Orientation (°)**	

		**Voltage DUT (V)**	**Voltage DUT (V)**	**Axis 1**	**Axis 2**	**Axis 1**	**Axis 2**
1	16	0.271417	0.158078	0.0031641	0.0019094	31.1089545	58.8910455
2	16	0.271323	0.157543	0.0031630	0.0019029	31.0318636	58.9681364
3	16	0.274602	0.156548	0.0032012	0.0018909	30.5694809	59.4305191
4	16	0.274084	0.158195	0.0031952	0.0019108	30.8803561	59.1196439
5	16	0.270761	0.15832	0.0031565	0.0019123	31.209151	58.790849
Average		0.2724374	0.1577368	**0.003176**	**0.001905**	**30.96**	**59.04**
uncertainty expanded				**0.000091**	**0.000077**	**1.50**	**2.91**

**Table 6.** Data registry system and storage system characteristics.

**Parameter**	**Channel 1**	**Channel 2**	**Channel 3**	**Channel 4**
THD (harmonics+noise)(dB)	−108.4	−112	−112	−112.4
THD (harmonics+noise) (%)	3.79 × 10^−4^	2.52 × 10^−4^	2.51 × 10^−4^	2.39 × 10^−4^
THD (dB)	−125.4	−124.3	−125.1	−126.9
THD (%)	5.39 × 10^−5^	6.09 × 10^−5^	9.94 × 10^−5^	4.51 × 10^−5^
SINAD (dB)	109.5	114	113.4	114
SNR (dB) (input: 20 μVpp)	18.7	21.44	21.43	21.58
DR (dB)	128	129.7	129.8	129.8
ENOB (bits)	21	21.44	21.43	21.56
Random noise (LSB) (4 active channels)	1.125	1.13	1.175	1.156
Crosstalk (dB)	−147.28	−147.4	−149.4	−145.4
Crosstalk (%)	4.33 × 10^−6^	4.28 × 10^−6^	3.38 × 10^−6^	5.35 × 10^−6^
Jitter (ns)	78.00 ± 3.40	76.53 ± 3.34	76.17 ± 3.32	76.57 ± 3.32
Time stability	2.873 × 10^−11^@108 h			
Allan deviation				
Clock drift	44 ms /day			
Memory capacity	2 GB			
Autonomy	2 month			

## References

[1] Rodríguez I., Mánuel A., Carlosena A, Bermúdez A., del Río J, Shariat-Panahi S. (2006). A signal processing in ocean bottom seismographs for refraction seismology. IEEE Trans. Instrum. Meas.

[2] Strömberg J.-O., Coifman A., Vassiliou A.Z., Averbuch R., Meyer F. (2001). Low bit-rate efficient compression for seismic data. IEEE Trans. Image Process.

[3] Dorman J., Ewing M. (1962). Numerical Inversion of seimic surface wave dispersion data and crust mantle structure in the New York-Pennsylvania Area. J. Geophys. Res.

[4] Azañón J.M., Azor A., Alonso F.M., Orozcoy M. (2002). Geología Física.

[5] Gaeta M., Briolle F., Esparcieux P. Blind separation of sources applied to convolutive mixtures in shallow water.

[6] Zhang X., Zhang A., Fang J., Yang S. Signal Study on Blind Separation of Underwater Acoustic Signals.

[7] Boles P.J., Boashash B., Boashash B. (1992). Applications of the cross-Wigner-Ville distribution to seismic data processing. Time–Frequency Signal Analysis: Methods and Applications.

[8] Setayeshi S., El-Hawary F., El-Hawary M.E. Underwater signal prediction and parameter estimation using artificial neural networks.

[9] Bormann P. (2002). NMSOP New Manual of Seismological Observatory Practice.

[10] Roset X., Carbonell M., Mànuel A., Gomáriz S. Sea seismometer coupling on the sediment.

[11] Shariat-Panahi S., Alegria F., Mànuel A. (2009). Design and test of a high resolution acquisition system for marine seismology. IEEE I&M Mag.

[12] Sheriff R.E., Geldarty L.P. (1995). Exploration Seismology.

[13] Roset X., Mànuel R., Palomera A. (2004). Contributions to Model and Characterization of Geophone Sensor.

[14] Brooks D. (2003). Signal Integrity Issues and Printed Circuit Board Design.

[15] Zang M., Cao W. (2009). A 0.1 ppm successive approximation frequency-temperature compensation method for Temperature Compensated Crystal Oscillators (TCXO).

[16] Shioda T., Sekine Y., Otsuka H. High precision TCXO for rapid environmental temperature change.

[17] Zelenka J., Lee P. Frequency temperature characteristics of the x-length strip resonators of AT-Cut quartz.

[18] Filler R., Rosati V., Schodowk S., Vig J. Specification and measurement of the frequency *versus* temperature characteristics of crystal oscillators.

[19] Aguzzi J., Mànuel A., Condal F., Guillén J., Nogueras M., del Rio J., Costa C., Menesatti P., Puig P., Sardà F. (2011). The new Seafloor Observatory (OBSEA) for remote and long-term coastal ecosystem monitoring. Sensors.

[20] Del Rio J., Toma D., Mànuel A., Ramos H. Evaluation of IEEE1588 applied to synchronized acquisition in marine sensor networks (MSN).

[21] Michaud F., Dañobeitia J.J., Carbonell R., Bartolomé R., Córdoba D., Delgado-Argote L. (2000). New insights about the oceanic crust entering the Middle American Trench off western Mexico (17–19° N). Tectonophysics.

[22] Shariat Panahi S., Ventosa S., Cadena J., Mànuel-Làzaro A., Bermudez T., Sallares V., Piera J. (2008). A low power datalogger based on CompactFlash memory for ocean bottom seismometers (OBS). IEEE Trans. Instrum. Meas.

[23] Input-Output Inc. Stafford, TX, USA. Available online: www.iongeo.com (accessed March 2012).

[24] Geo-Space.

